# Supplementary figures and images for: Metformin Alleviates Left Ventricular Diastolic Dysfunction in a Rat Myocardial Ischemia Reperfusion Injury Model
Source: Int J Mol Sci. 2020 Feb 21;21(4):1489. doi: 10.3390/ijms21041489 (PMC7073047; doi:10.3390/ijms21041489)

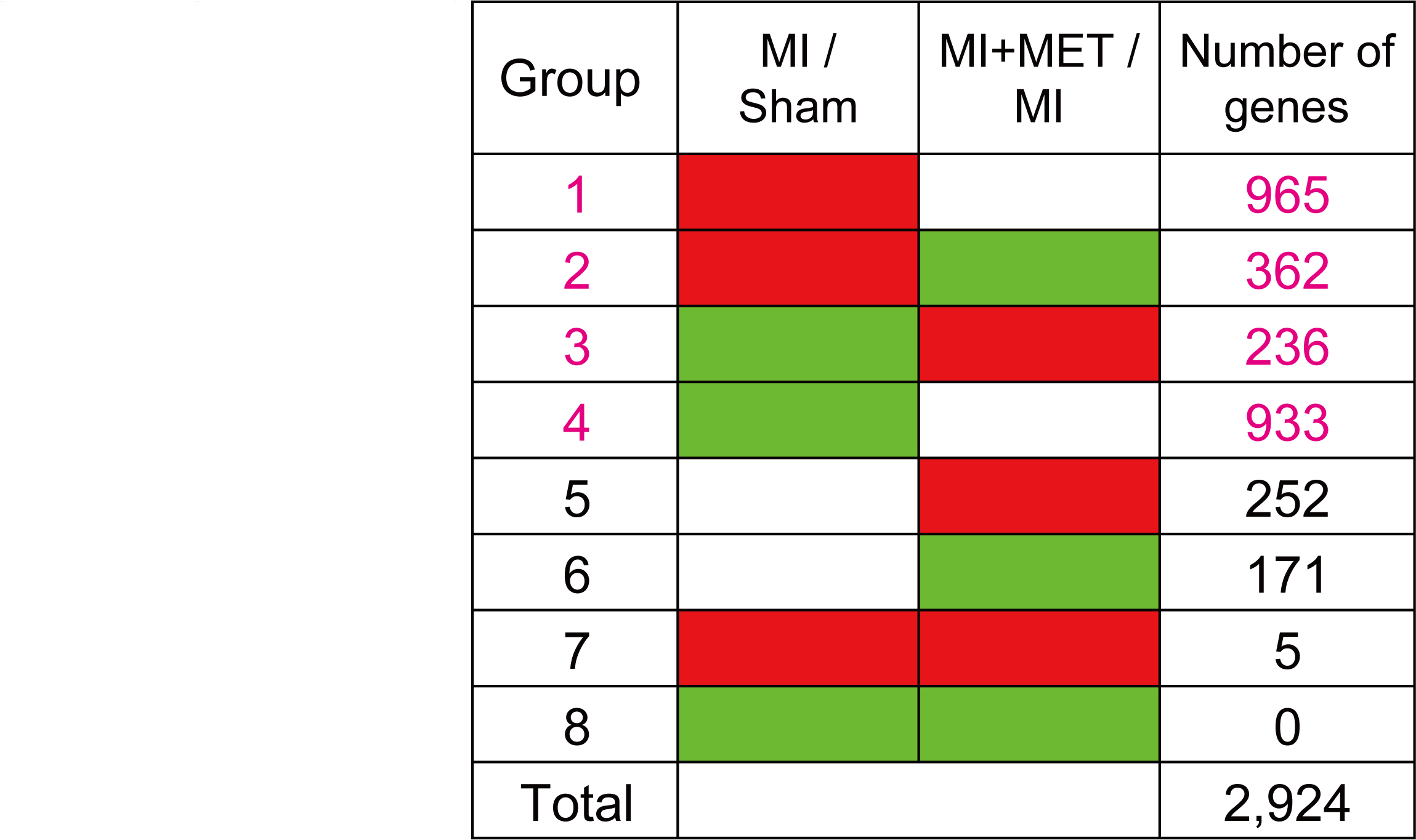

Supplement: Supplementary file 1 [file ijms-21-01489-s001.zip › Supplementary files_revised_/Supplementary FigureS1.tif]
