# Supplementary material for: Metformin Alleviates Left Ventricular Diastolic Dysfunction in a Rat Myocardial Ischemia Reperfusion Injury Model
Source: Int J Mol Sci. 2020 Feb 21;21(4):1489. doi: 10.3390/ijms21041489 (PMC7073047; doi:10.3390/ijms21041489)
Supplement: Supplementary file 1 [file ijms-21-01489-s001.zip › Supplementary files_revised_/Supplementary Materials.doc]

**Supplementary Materials**

**Metformin Alleviates Left Ventricular Diastolic Dysfunction in a Rat Myocardial Ischemia Reperfusion Injury Model**

Woori Jo, Kyung-Ku Kang, Sehyun Chae, and Woo-Chan Son

**Supplementary Figure**

**Figure S1.** 8 clusters of the DEGs. Colors represent the increase (red) and decrease (green) of mRNA expression levels. The number of the genes in each cluster was shown. Groups 1-4 with the four largest sizes were defined as four major groups in Figure 4B.

**Supplemental Tables**

**Table S1.** Individual histopathology scorings are shown. Grading of histopathological changes in the rat left ventricle SAX with papillary muscle and apex area tissue. Grades 1, 2, 3 and 4 show minimal, slight, moderate, and severe pathological changes, respectively.

|  | MI | | | | | | | | | | MI+MET | | | | | | | | | | |
| --- | --- | --- | --- | --- | --- | --- | --- | --- | --- | --- | --- | --- | --- | --- | --- | --- | --- | --- | --- | --- | --- |
|  | Apex | | | | | SAX with papillary muscle | | | | | Apex | | | | | | SAX with papillary muscle | | | | |
| Lesions | 1 | 2 | 3 | AVG | SD | 1 | 2 | 3 | AVG | SD | | 1 | 2 | 3 | AVG | SD | 1 | 2 | 3 | AVG | SD |
| Coagulation necrosis, myocardial | 3 | 4 | 3 | 3.33 | 0.58 | 3 | 4 | 3 | 3.33 | 0.58 | | 3 | 2 | 3 | 2.67 | 0.58 | 3 | 2 | 3 | 2.67 | 0.58 |
| Inflammatory cells infiltration, epicardial | 3 | 2 | 2 | 2.33 | 0.58 | 4 | 2 | 2 | 2.67 | 1.15 | | 2 | 1 | 3 | 2.00 | 1.00 | 1 | 2 | 2 | 1.67 | 0.58 |
| Inflammatory cells infiltration, myocardial | 3 | 4 | 3 | 3.33 | 0.58 | 4 | 4 | 3 | 3.67 | 0.58 | | 3 | 2 | 3 | 2.67 | 0.58 | 4 | 3 | 3 | 3.33 | 0.58 |
| Inflammatory cells infiltration, endocardial | 3 | 2 | 2 | 2.33 | 0.58 | 2 | 2 | 1 | 1.67 | 0.58 | | 1 | 1 | 2 | 1.33 | 0.58 | 2 | 1 | 1 | 1.33 | 0.58 |
| Fibrosis, epicardial | 4 | 2 | 2 | 2.67 | 1.15 | 4 | 1 | 2 | 2.33 | 1.53 | | 3 | 1 | 3 | 2.33 | 1.15 | 2 | 1 | 3 | 2.00 | 1.00 |
| Fibrosis, myocardial | 4 | 4 | 3 | 3.67 | 0.58 | 4 | 4 | 3 | 3.67 | 0.58 | | 3 | 2 | 3 | 2.67 | 0.58 | 2 | 2 | 3 | 2.33 | 0.58 |
| Fibrosis, endocardial | 3 | 2 | 2 | 2.33 | 0.58 | 4 | 1 | 1 | 2.00 | 1.73 | | 1 | 1 | 3 | 1.67 | 1.15 | 1 | 1 | 1 | 1.00 | 0.00 |
| Total |  |  |  | 2.86 | 0.57 |  |  |  | 2.76 | 0.81 | |  |  |  | * 2.19 | 0.54 |  |  |  | 2.05 | 0.80 |

* *p* < 0.05 indicates a statistically significant difference by two-tailed unpaired t-test compared with the MI group.

**Table S2.** 2,924 DEGs. For each DEG, gene symbol and description, adjusted P-value, log2-fold-change of mRNA levels between two comparisons are shown. The genes in cluster 1-8 are also denoted.

See the attached excel file.
